# Phenotypicand Genotypic Characterization of Clinical Isolates of Intracellular Adherent–Invasive *Escherichia coli* Among Different Stages, Family History, and Treated Colorectal Cancer Patients in Iran

**DOI:** 10.3389/fcimb.2022.938477

**Published:** 2022-07-11

**Authors:** Razie Kamali Dolatabadi, Hossein Fazeli, Mohammad Hassan Emami, Vajihe Karbasizade, Fatemeh Maghool, Alireza Fahim, Hojatollah Rahimi

**Affiliations:** ^1^ Department of Microbiology, School of Medicine, Isfahan University of Medical Sciences, Isfahan, Iran; ^2^ Poursina Hakim Digestive Diseases Research center, Isfahan University of Medical Sciences, Isfahan, Iran

**Keywords:** adherent–invasive *Escherichia coli*, colorectal cancer, cyclomodulins, virulence factors, rep-PCR

## Abstract

There is increasing evidence showing that microbial dysbiosis impacts the health and cancer risk of the host. An association between adherent–invasive *Escherichia coli* (AIEC) and colorectal cancer (CRC) has been revealed. Cyclomodulins (CMs) have been receiving increasing attention for carcinogenic changes. In this study, the incidence and features of intracellular AIEC and cyclomodulin-encoding genes were investigated and the phylogenetic grouping and genetic relatedness were evaluated. *E. coli* strains were isolated from the colorectal biopsies. Adhesion and invasion assays and intramacrophage cell survival test were performed to separate the AIEC isolates. Virulence genotyping for the genes *htrA*, *dsbA*, *chuA*, and *lpfA* and the cyclomodulin toxins was also conducted. In addition, phylogenetic grouping of the isolates was determined. Subsequently, repetitive element sequence-based PCR (rep-PCR) fingerprinting was performed. A total of 24 AIEC pathovars were isolated from 150 patients. The prevalence rates of *htr*, *dsbA*, and *lpfA* were 70.83% and that of *chuA* was 91.66%. The frequencies of the cyclomodulin toxins were as follows: *cnf1*, 29.2%; *cnf2*, 25%; colibactin, 29.2%; and *cdt*, 4.2%; *cif* was not found. Among the AIEC isolates, 4.2%, 4.2%, 54.2%, 29.2%, and 8.3% with phylotypes A or C, B1, B2, D, and E were identified, respectively. Left-sided colon carcinoma and adenocarcinoma T≥1 stage (CRC2) were colonized by B2 phylogroup AIEC-producing CMs more often than the samples from the other groups. Close genetic relatedness was observed in AIEC isolates with rep-PCR.

## Introduction

According to the World Health Organization, infections are responsible for about 25% of all malignancies. Colorectal cancer (CRC) is the third most prevalent cancer and the second most lethal disease, with an expected 1.9 million diagnoses and 0.9 million deaths worldwide in 2020 (Xi and Xu, 2021). Approximately 95% of CRC cases are sporadic (Kwak and Chung, 2007). The American Joint Committee on Cancer (AJCC) TNM method is the most commonly used staging system for CRC. It is entirely based on three important items: the extent (size) of the tumor (T), the involvement of regional lymph nodes (N), and metastasis to distant sites (M) (Edition et al., 2017).

Approximately 20% of all CRCs are attributed to genetic factors, with the remaining 80% being caused by environmental factors, such as microorganisms in the colonic ecosystem (Half et al., 2009). There is increasing evidence showing that the composition of the human gut microbiota impacts the health of the host. Microbial dysbiosis was observed in CRC patients, with evidence of bacterial cooperation for *Bacteroides fragilis*, *Helicobacter pylori*, *Streptococcus bovis*, and *Enterococcus* spp. being documented (Collins et al., 2011). An association between adherent–invasive *Escherichia coli* (AIEC) and CRC has been discovered in several investigations. Mucosa-associated *E. coli* is more frequently found in colon tissue from patients with adenocarcinoma than in controls, according to previous investigations (Martin et al., 2004; Camprubí-Font et al., 2020). *E. coli* is the most common Gram-negative species of the human gut normal flora that participates in preserving and maintaining the stability of the intestinal bacterial flora and gut homeostasis (Buc et al., 2013). Because of good harmony with its human host, *E. coli* rarely causes diseases. However, a number of strains, such as pathogenic *E. coli* (PEC), possess a mixture of virulence genes that allow them to induce intestinal (InPEC) and extraintestinal (ExPEC) infections (Kaper et al., 2004; Croxen and Finlay, 2010). One of the diagnosed *E. coli* pathovars (found in the late 1990s.), AIEC, is in this transfer spectrum. *E. coli* strains with comparable pathogenic functions to AIEC are associated with intestinal problems along with inflammatory bowel diseases (IBDs), ulcerative colitis, and Crohn’s disease, as well as CRC. However, the prevalence of AIEC in these illnesses remains in large part unexplored. Regarding the lack of virulence factors, AIEC is different from other pathogenic type *E. coli*, such as Shiga toxin-producing *E. coli* (STEC), enteropathogenic *E. coli* (EPEC), enteroinvasive *E. coli* (EIEC), diffusely adherent *E. coli* (DAEC), enteroaggregative *E. coli* (EAEC), and enterotoxigenic *E. coli* (ETEC) (Croxen et al., 2013). Since AIEC was first described a decade ago, significant progress has been made to understand its pathogenic mechanisms (Martinez-Medina and Garcia-Gil, 2014). Because AIEC isolates lack the characteristic *E. coli* virulence factors, they are phenotypically characterized *via* their functionality of adhesion and invasion of intestinal epithelial cells. The presence of carcinoembryonic antigen in the apical area of epithelial cells, as well as the expression of type 1 pili surrounding the surface of the bacterial cell and through cell adhesion molecule 6 (CEACAM6), is thought to be responsible for this form of attachment (Chervy et al., 2020; Abdelhalim et al., 2020a). Other characteristics of this pathovar include surviving and replicating in macrophages by secreting a large amount of tumor necrosis factor alpha without inducing death of the host cells. Some of the features of these *E. coli* are related to specific genes, including high temperature requirement A (*htrA*), oxidoreductase disulfide bond A (*dsbA*), and a reduction in iron uptake (*chuA*), which increases macrophage cell survival, and long polar fimbriae (*lpfA*), which causes microfold (M) cells to pass through the associated follicle epithelium (Chassaing et al., 2011; Dogan et al., 2014; Abdelhalim et al., 2020b). Phagolysosomes can lead to the continuous secretion of cytokines and chronic activation of macrophages (Glasser et al., 2001; Bringer et al., 2007). Among the virulence factors of *E. coli*, a number of genotoxic toxins called cyclomodulins (CMs) are receiving increasing attention because they regulate cell differentiation, apoptosis, and proliferation (Collins et al., 2011). The activation of Rho-GTPases by cytotoxic necrotizing factor (*cnf*) causes alterations in the cytoskeleton and affects the cell cycle. The cycle-inhibiting factor (*cif*) hijacks host cell signaling pathways by targeting NEDD8-conjugated cullins (Mannion et al., 2022). The genotoxin colibactin is a hybrid polyketide–non-ribosomal peptide. Its biosynthetic mechanism is encoded by the genomic island *pks*. Colibactin causes DNA double-strand breaks and chromosomal instability in human eukaryotic cells (Iwasaki et al., 2022; Mezerova et al., 2022). Cell-lethal distending toxins (*cdts*) induce DNA damage, presumably *via* DNAse activity, and are closely related enzymes produced by *Helicobacter hepaticus* that progress to precancerous dysplastic lesions of hepatitis. The cytolethal distending toxin (*cdt*) causes DNA damage and provides evidence of its carcinogenic potential in humans (Bennedsen et al., 2022).

The incidence and the features of intracellular AIEC, genotoxin- and cyclomodulin-encoding genes, and the phylogenetic grouping and genetic relatedness were evaluated in isolates from colorectal tissue samples of CRCs (adenocarcinoma *in situ* and tumor stage T1, T2, or T3). In addition, the family history of patients with CRC, patients with a history of CRC, and controls were studied.

## Materials and Methods

### Patients and Tissue Samples

The *E. coli* strains were obtained from the colorectal biopsies of Iranian patients who were referred to the Poursina Hakim Digestive Diseases Research Center and Clinic, Isfahan University of Medical Sciences, Isfahan, Iran, for suspected colorectal neoplasia from June 2019 to February 2022. The patients included in this study ranged in age from 25 to 92 years, they did not have any other inflammatory disease in the colon, and they had been without antimicrobial treatment for at least 1 month. All participants signed written informed consent before enrolment. This study was approved by the Ethics Committee of the Isfahan University of Medical Sciences (approval no. IR.MUI.MED.REC.1398.319).

Colorectal tissue samples were taken from 150 patients at the time of colonoscopy by experienced endoscopists. Biopsies were collected using sterile forceps, immediately deposited in sterile tubes for *E. coli* isolation with 0.9% normal saline, and maintained at 4°C (Camprubí-Font et al., 2019). The biopsy specimens were processed according to a standard methodology for pathological examination. For CRC staging, the TNM classification method was employed. The samples were classified into five groups based on clinicopathological features: 30 patients with normal colonoscopy results (control group); 30 patients with normal results of colonoscopy, but with a family history of CRC based on the National Comprehensive Cancer Network (FH group); 30 patients with normal colonoscopy results who had a personal history of CRC referred for a 6-month follow-up (CRChis group); and a group of 60 patients with adenocarcinoma, of which 30 patients were reclassified into stage 0 adenocarcinoma *in situ* (Tis) (CRC1 group) and the other 30 patients with tumor stage T1, T2, or T3 (CRC2 group).

**Supplementary Table S1** lists the clinical features of the individuals who took part in the trial. The mean (range) ages were 55.03 (30–82) years for the CRC1 group, 63.17 (26–92) years for the CRC2 group, 53.74 (25–82) years for the FH group, 55.81 (28–69) years for the CRChis group, and 57.71 (35–73) years for the controls.

### Sample Treatment

With slight modifications, *E. coli* was isolated from the biopsy specimens based on the method described by Céspedes et al. (2017). Each biopsy sample was washed in buffered physiological saline supplemented with 0.016% dithioerythritol (DTT) (Solarbio, Beijing, China) to remove the mucus and then rinsed in buffered physiological saline. The sample was then placed in 100 µg/ml gentamicin (HiMedia Co., Mumbai, India) solution and incubated at 37°C for 1 h to suppress extracellular bacteria. Biopsies were washed three times with phosphate-buffered saline (PBS) and lysed with 100 µl of 1% Triton-X to extract intracellular bacteria (Céspedes et al., 2017). The cell lysis solution was immediately cultured on MacConkey agar plate and aerobically incubated at 37°C overnight. Grown colonies were examined using Analytical Profile Index (API) biochemical tests (API 10S-ZIPAS, Iran), including *ortho*-nitrophenyl-β-galactoside (ONPG), indole (IND), glucose (GLU), lysine decarboxylase (LDC), citrate (CIT), urease (URE), tryptophan deaminase (TDA), arabinose (ARA), ornithine decarboxylase (ODC), oxidase, H_2_S, and NO_2_ reaction. The *E. coli* isolates were determined using standard identification tables. After confirming the phenotype of the isolates, the *uid* gene (beta-glucuronidase) was amplified using polymerase chain reaction (PCR) to finally identify the genus and species of *E. coli* (Mahon et al., 2018).

### Culture of Caco-2 and J774A.1 Cells

Human colorectal adenocarcinoma (Caco-2) cells (code NCBI-C10094, Iran) and the murine macrophage-like cell line J774A.1 were prepared by the National Cell Bank of Iran (code NCBI-C483, Iran). Caco-2 cells were used to determine the adhesion and invasion ability of the *E. coli* isolates. The murine macrophage-like cell line J774A.1 was utilized to investigate the survival and replication inside macrophage cells. The cells were cultured in Dulbecco’s modified Eagle’s medium (DMEM) supplemented with 10% fetal bovine serum (Bio-idea, Tehran, Iran) and 1% antibiotics, then incubated at 37°C in a humidified environment with a humidity of 95% and a CO_2_ concentration of 5% (INC246; Memmert, Schwabach, Germany). Further studies were conducted using logarithmically active cells.

### Adhesion and Invasion Assays

To evaluate the adherence and invasive potency of the isolates, the gentamicin protection assay of Darfeuille Michaud et al. (2004) was used, with a few modifications (Darfeuille-Michaud et al., 2004).

The capacity to adhere to intracellular *E. coli* isolates was examined. Monolayers of Caco-2 cells with a density of 5 × 10^5^ cells/well were seeded on a 24-well plate. *E. coli* isolates in the exponential phase were harvested and added to DMEM without antibiotics. Caco-2 cells were inoculated with 0.5 ml bacterial suspension [10^6^ bacteria/ml, a multiplicity of infection (MOI) of 10] and then incubated for 3 h in 5% CO_2_. Subsequently, the infected monolayer was thoroughly washed with PBS three times, and the cell monolayer was lysed with 1% Triton X-100 to obtain adherent bacteria. Lysed cells were then diluted stepwise and cultured in brain heart infusion (BHI) medium. The number of adherent bacteria was calculated by dividing the proportion of adhering bacteria by the total number of bacteria acquired following infection. All of the tests were carried out three times (da Motta Willer et al., 2004). The cell invasion assay was performed. Adherent-positive strains were used to infect a 24-well plate of Caco-2 monolayer at a density of 5 × 10^5^ cells/well. A monolayer of the Caco-2 cell line was infected using 0.5 ml of bacterial suspension (10^6^ bacteria/ml, MOI = 10) and incubated for 3 h in 5% CO_2_. The infected monolayer cells were washed three times with PBS after incubation, and each well was supplemented with fresh DMEM containing 100 µg/ml gentamicin and incubated for 1 h to grow intracellular bacteria. When the incubation period was over, the cells were thoroughly washed with PBS three times and then lysed with 1% Triton X-100. The diluted lysates were cultured on BHI agar medium and then the number of viable (invasive) bacteria measured. When the intracellular bacteria/primary inoculum number × 100 ratio of an *E. coli* isolate was 0.1% or higher, it was referred to as invasive. All assays were repeated three times (Etienne-Mesmin et al., 2011). *E. coli* K12 (IBRCM11152) and *Shigella sonnei* (IBRCM10672) were the negative and positive controls, respectively (da Motta Willer et al., 2004).

### Intramacrophage Cell Survival Test

Survival assays were performed as defined according to Glasser et al. (2001), with a few modifications. Briefly, the murine macrophage cell line J774A.1 was seeded in a 24-well plate at a density of 1 × 10^5^ cells/well, followed by infection with an *E. coli* isolate (1 × 10^6^ bacteria/ml, MOI = 10). The cells were incubated for 3 h at 37°C in a 5% CO_2_ environment. The medium was extracted after incubation, the macrophage cells washed twice with PBS, and the wells were replaced with fresh medium containing 100 µg/ml gentamicin to remove extracellular bacteria. After extraction of the medium and replenishing with fresh medium containing 50 µg/ml gentamicin, the infected monolayer was cultured for another 24 h. Subsequently, the cells were washed in sterile PBS, lysed with 1% Triton X-100, and the lysates diluted and grown on trypticase soy agar (TSA) medium. The ratio of the number of intracellular bacteria obtained after 1 h of incubation to the number of intracellular bacteria obtained after 24 h was calculated to estimate the level of survival. All assays were performed in triplicate (Glasser et al., 2001). Strains *E. coli* K12 (IBRC-M11152) and *Salmonella enterica* subsp*. Enterica* (IBRC-M10957) were used as negative and positive controls, respectively (Etienne-Mesmin et al., 2011). The adherent-positive strains with INV% > 0.1 and REPL% = 100% were considered as AIEC strains in the study.

### Virulence Genotyping

For DNA extraction, each strain was cultured in a broth medium for 24 h at 37°CC. Genomic DNA was extracted as previously described (Hsu et al., 1993).

For virulence genotyping, several genes that are known to play a role in bacterial survival in macrophages (*htrA*, *dsbA*, and *chuA*), transmission agents from M and follicle-associated epithelium (FAE) cells (*lpfA*), and cyclomodulin genotoxic toxins that modulate cell differentiation, apoptosis, and proliferation (*cnf*, *cif*, *colibactin*, and *cdt*) were examined. The PCR primer sequences are shown in **Supplementary Table S2**.

### Phylogenetic Grouping

Using a multiplex PCR approach, all intracellular *E. coli* isolates were classified into phylogroups A, C, B1, B2, D, E, and F (Clermont et al., 2013).

### (GTG) 5-Rep-PCR Fingerprinting

All AIEC isolates were selected for analysis with repetitive element sequence-based PCR (rep-PCR). The primer 5′-GTGGTGGTGGTGGTG-3′ was employed to amplify the chromosomal DNA repetitive regions of the AIEC isolates. Based on the manufacturer’s instructions, PCR was performed using a commercially stocked PCR Master Mix (Ampliqon, Odense, Denmark). Briefly, 0.3 µl of the primer (10 pmol/µl), 6 µl of the Master Mix, 5.2 µl of DNase-free distilled water, and 1 µl template DNA were mixed in a final volume of 12.5 µl. Amplification was performed in a 48-well T100 Thermal Cycler (Bio-Rad, Hercules, CA, USA) as follows: initial denaturation at 94°C for 5 min, followed by 30 cycles of denaturation at 94°C for 30 s, annealing at 45°C for 1 min, extension at 65°C for 8 min, and a final extension at 65°C for 16 min (Khare et al., 2020).

### PCR Product Analysis

The PCR products were electrophoresed on 1.5% agarose gel using 1× Tris–borate–EDTA (TBE) buffer at 65 V for 2 h on 1.5% agarose gel. The experiment was carried out a total of three times. As in a previous study, the rep-PCR patterns were examined using GelJ software (Heras et al., 2015). Isolates were classified together into the same genotype if their similarity coefficient was 80% or greater.

### Statistical Analysis

Fisher’s exact test and the chi-square test were used for statistical analysis. Firstly, the chi-square test for heterogeneity was used for multiple-group comparisons. At *p* < 0.05, differences were considered to be significant. IBM SPSS Statistics 24.0 was used for all statistical methods.

## Results

### Characteristics of the Subjects

The most common indications in the study participants were constipation (41.17%) in the CRC1 group, a positive fecal immunochemical test (FIT) or hematochezia (50%) in the CRC2 group, screening (53.33%) in the FH group, screening and a positive FIT (each 23.33%) in the control group, and follow-up of CRC history (90.4%) in the CRChis group (**Table 1**).

**Table 1:** Characteristics of the study subjects.

Groups	Mean age (years)	Gender		Indication								
		Male	Female	Constipation	FIT-positive or hematochezia	Lower abdominal pain, dyspepsia	Iron deficiency	Screening	Follow-up of CRC history	Chronic diarrhea	FH of CRC	ND
CRC1	55.03	19 (63.33%)	11 (36.67%)	14 (46.67%)	9 (30%)	4 (13.33%)	1 (3.33%)	–	–	–	2 (6.67%)	–
CRC2	63.17	14 (46.67%)	16 (53.33%)	3 (10%)	15 (50%)	4 (13.34%)	1 (3.33%)	–	2 (6.67%)	2 (6.67%)	1 (3.33%)	2 (6.67%)
CRChis	55.81	18 (60%)	12 (40%)	–	–	–	3 (10%)	–	27 (90%)	–	–	–
FH	53.74	17 (56.67%)	13 (43.33%)	5 (16.67%)	5 (16.67%)	–	3 (10%)	15 (50%)	–	2 (6.67%)	–	–
Control	57.71	11 (36.67%)	19 (63.33%)	9 (30%)	7 (23.33%)	7 (23.33%)	–	7 (23.33%)	–	–	–	–

FIT, fecal immunochemical test; ND, no data; CRC1, adenocarcinoma colorectal cancer in situ (Tis); CRC2, colorectal cancer with tumor stage T1, T2, or T3; CRChis, with CRC history, but normal colonoscopy results, who have been referred for a 6-month follow-up; FH, normal results on colonoscopy, but with a family history of CRC based on the National Comprehensive Cancer Network.

### Characteristics of the Isolated Intracellular *E. coli*

A total of 74 intracellular *E. coli* were isolated from 150 patients: 18 isolates from the CRC1 group, 20 from the CRC2 group, 14 from the CRChis group, 11 from the FH group, and 11 isolates from the control group. The prevalence of intracellular *E. coli* did not differ significantly among the groups studied (*p* = 0.063). The majority of intracellular *E coli* were isolated from rectal specimens (31/74, 41.9%), followed by sigmoid specimens (14/74, 18.9%). Significant differences in the prevalence of intracellular *E. coli* isolated from the sampling locations (*p* = 0.004) were observed (**Table 2**).

**Table 2:** Prevalence of the *Escherichia coli* isolates.

		Number (%)	*p*-value
Studied group	CRC1	18 (24.3)	0.063
	CRC2	20 (27)	
	CRChis	14 (18.9)	
	FH	11 (14.9)	
	Control	11 (14.9)	
Sampling location	Transverse colon	8 (10.8)	0.004
	Ascending colon	2 (2.7)	
	Cecum	9 (12.2)	
	Ileum	0	
	Hepatic flexure	3 (4.1)	
	Descending colon	7 (9.5)	
	Sigmoid	14 (18.9)	
	Rectum	31 (41.9)	

The prevalence of the E. coli isolates by groups and sampling location was calculated using the chi-square test.

CRC1, adenocarcinoma colorectal cancer in situ (Tis); CRC2, colorectal cancer with tumor stage T1, T2, or T3; CRChis, with CRC history, but normal colonoscopy results, who have been referred for a 6-month follow-up; FH, normal results on colonoscopy, but with a family history of CRC based on the National Comprehensive Cancer Network.

There were significant differences in the proportions of adherent and invasive bacteria among the 74 intracellular *E. coli* isolates from the analyzed groups, indicating that the median of these isolates differed in groups. No significant differences in the intramacrophagic survival and replication capacities (the ability to survive and multiply inside macrophages) were observed in the tested groups, as shown in **Table 3**.

**Table 3:** Characteristics of the isolated adherent–invasive *Escherichia coli* (AIEC) strains.

Characteristics		ADH^a^		INV^b^ (%)		REPL^c^ (%)	
		Mean ± SD	*p*-value	Mean ± SD	*p*-value	Mean ± SD	*p*-value
Reference strain	*E. coli* K-12	0.63 ± 0.05	–	0.04 ± 0.03	–	25.09 ± 4.34	–
	*Shigella sonnei* (ATCC 9290)	3.64 ± 1.13		0.49 ± 0.304	–	–	–
	*Salmonella enterica* (ATCC 9270)	–	–	–	–	213.512 ± 21	–
Disease group	Control	1.603 ± 0.550	0.007 (S)	0.134 ± 0.029	0.006	139.646 ± 18.49	0.037
	CRC1	1.902 ± 0.397		0.258 ± 0.190		304.342 ± 142.42	
	CRC2	2.138 ± 0.810		0.540 ± 0.143		464.573 ± 227.204	
	CRChis	1.338 ± 0.224		0.262 ± 0.142		229.554 ± 174.462	
	FH	1.054 ± 0.460		0.317 ± 0.067		157.531 ± 26.636	

Data were analyzed using one-way ANOVA.

S, significant (*<0.05); NS, not significant for null (p > 0.05) association. CRC1, adenocarcinoma colorectal cancer in situ (Tis); CRC2, colorectal cancer with tumor stage T1, T2, or T3; CRChis, with CRC history, but normal colonoscopy results, who have been referred for a 6-month follow-up; FH, normal results on colonoscopy, but with a family history of CRC based on the National Comprehensive Cancer Network.

a Grade of adhesion.

b Percentage of inoculum surviving after 1 h of gentamicin treatment (number of intracellular bacteria/initial inoculum × 100).

c Number of intracellular bacteria at 24 h post-infection/number of bacteria at 1 h post-infection × 100 (%).

### Identification of AIEC

The AIEC isolates were analyzed using adhesion and invasion assays and the test of survival within macrophage cells based on the existing criteria described in the method used to define AIEC pathovar. A total of 24 AIEC pathovars were isolated from 74 intracellular *E. coli*: 7 (29.16%) in the CRC1 group, 8 (33.3%) in the CRC2 group, 4 (16.66%) from the CRChis group, 2 (8.3%) in the FH group, and 3 (12.5%) in the control group.

As can be seen, the characteristics of the AIEC isolates in the studied groups were significantly different from those of intracellular *E. coli* isolates. The highest invasion level was 0.540 ± 0.143, in the CRC2 group, while the lowest was 0.134 ± 0.029, in the control group. The highest survival level was 464.573 ± 227.204, in the CRC2 group, while the lowest was 139.646 ± 18.49, in the control group.

Between the study groups, there was a statistically significant difference in the mean level of adherence, invasion, and intramacrophage survival of AIEC isolates (**Table 3**).

### Phylogenetic Affiliation

**Figure 1:**
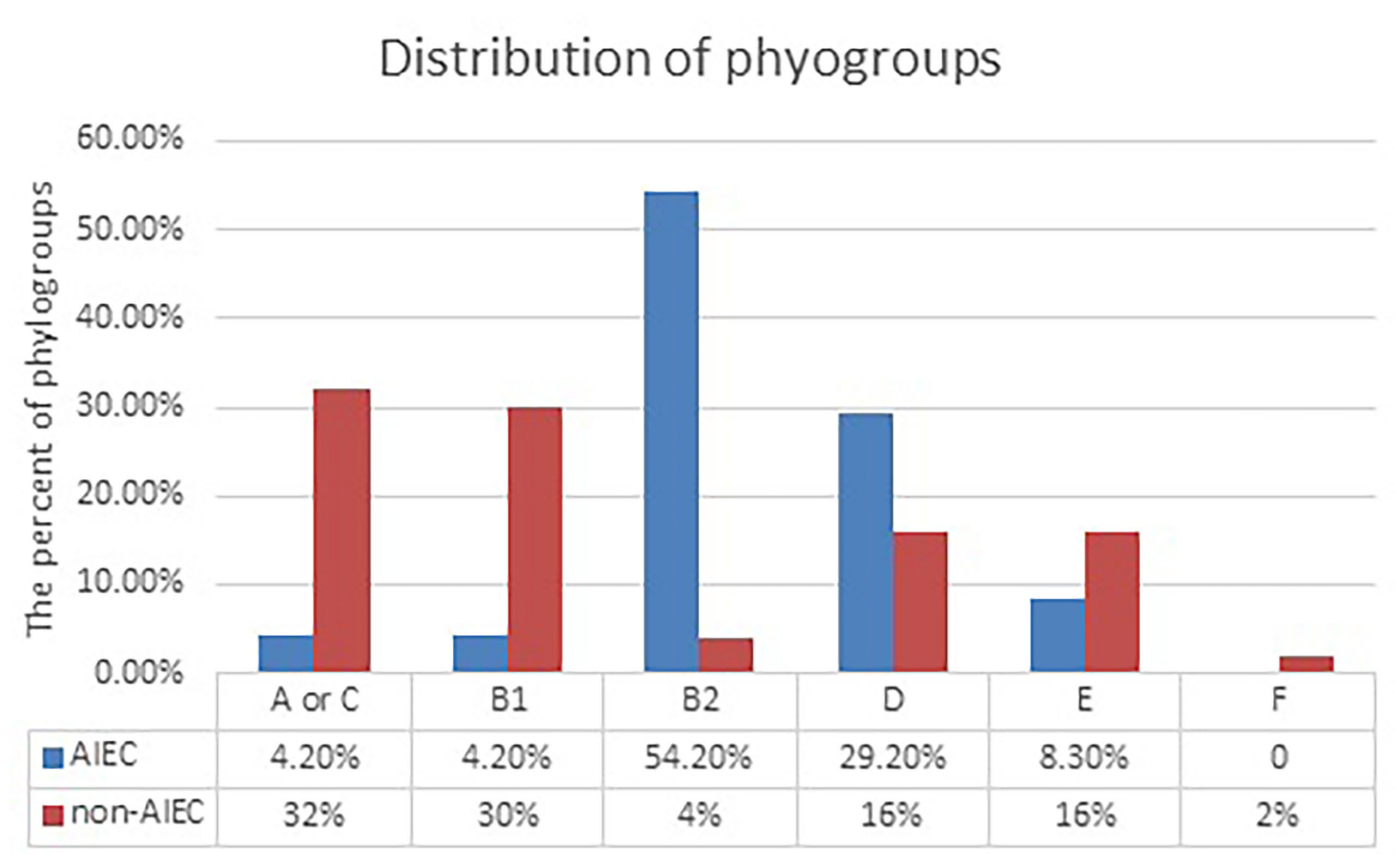
Distribution of adherent–invasive *Escherichia coli* (AIEC) and non-AIEC isolates according to phylogroups. Data were analyzed using chi- square test. *S*, significant (*<0.05); *NS*, not significant for null (*p* > 0.05) association.

The results of the analysis showed that the AIEC strains mostly belong to groups B2 and D, while the non-AIEC strains mostly belong to groups A or C and B1 (**Figure 1**). Of the 24 AIEC isolates, 1 (4.2%), 1 (4.2%), 13 (54.2%), 7 (29.2%), and 2 (8.3%) were identified as phylogroups A or C, B1, B2, D, and E, respectively. However, in the non-AIEC group, the frequencies of phylogroups A or C and B1 were higher (**Table 4**).

**Table 4:** Phylogenetic groups and virulence genes detected by PCR in *Escherichia coli* strains isolated from the study groups.

Groups and genes		%Frequencies (*n*)		*p*-value
		AIEC strain (*n* = 24)	Non-AIEC strain (*n* = 50)	
Toxins	*cnf1*	29.2% (7)	2% (1)	0.001
	*cnf2*	25% (6)	0	0.001
	*Colibactin*	29.2% (7)	0	<0.0001
	*cdt*	4.2% (1)	0	0.32
	*cif*	0	0	–
Virulence gene	*htrA*	70.8 (17)	0	<0.0001
	*dsbA*	70.8% (17)	0	
	*lpfA*	70.8% (17)	18% (9)	
	*chuA*	91.7% (22)	38% (19)	
Phylotype	A or C	4.2% (1)	32% (16)	<0.0001
	B1	4.2% (1)	30% (15)	
	B2	54.2% (13)	4% (2)	
	D	29.2% (7)	16% (8)	
	E	8.3% (2)	16% (8)	
	F	0	2% (1)	

Data were analyzed using the chi-square test.

S, significant (*<0.05*); NS, not significant for null (p > 0.05) association.

There was no significant difference in the mean of the percentage of adherent and invasive bacteria and in the quantification of the intramacrophage survival and replication abilities among the AIEC isolates according to phylogenetic grouping (**Table 4**).

### Virulence Genotyping

The prevalence rates of the virulence genes are shown in **Table 4**.

Of the AIEC isolates, the frequencies of CM toxins were as follows: *cnf1*, 7/24 (29.2%); *cnf2*, 6/24 (25%); colibactin, 7/24 (29.2%); and *cdt*, 1/24 (4.2%); *cif* was not found. However, 14 (54.16%) isolates contained at least one of the CM toxins. Of the AIEC strains isolated from patients with cancer, 92.3% carried one of the CM toxin genes, which was remarkable, whereas the AIEC strains isolated from the control and FH groups did not carry any CM toxin genes.

**Figure 2:**
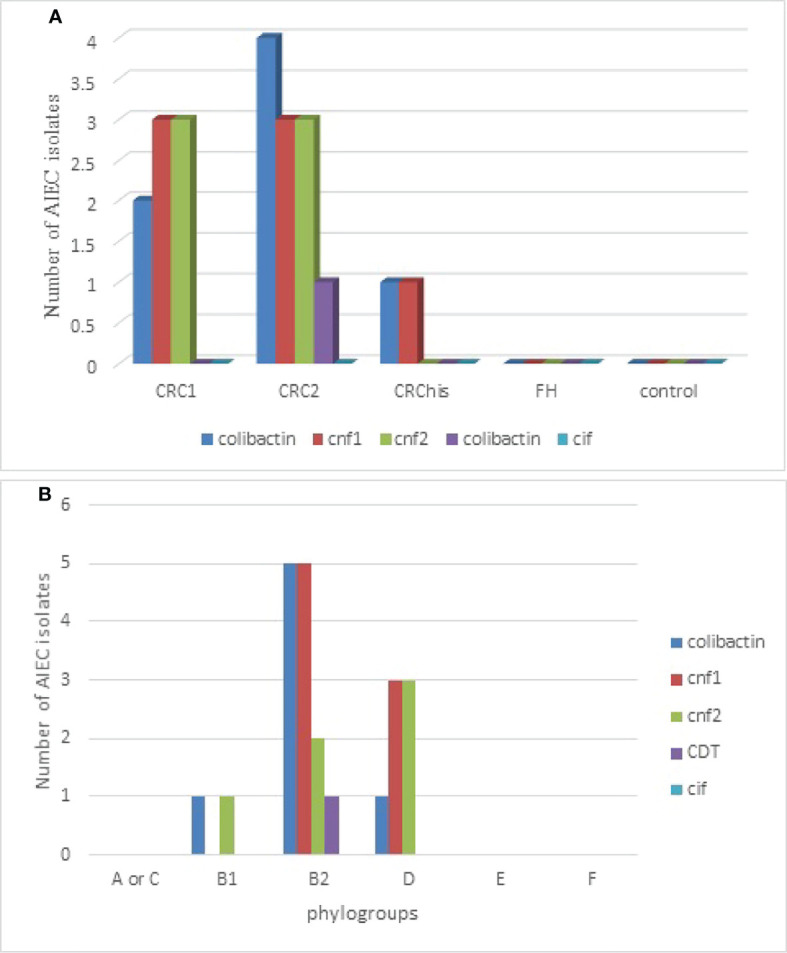
Distribution of adherent–invasive *Escherichia coli* (AIEC) isolates producing various cyclomodulins according to the studied groups **(A)** and to phylogroups **(B)**. *CRC1*, adenocarcinoma colorectal cancer *in situ* (Tis); *CRC2*, colorectal cancer with tumor stage T1, T2, or T3; *CRChis*, with CRC history, but normal colonoscopy results, who have been referred for a 6-month follow-up; *FH*, normal results on colonoscopy, but with a family history of CRC based on the National Comprehensive Cancer Network.

As shown in **Figure 2**, of the studied groups, the cancer groups, especially those in more advanced stages (CRC2), had the highest number of carcinogenic toxins.

Four genetic elements (i.e., *htr*, *dsbA*, *lpfA*, and *chuA*) have been suggested as PCR-tracked putative AIEC molecular markers. The prevalence rates of *htr*, *dsbA*, and *lpfA* were all 70.83% and that of *chuA* was 91.66%.

As shown in **Supplementary Table S4**, a significant relationship was observed between the survival levels and the presence of *dsbA* virulence factors. There was no statistically significant relationship between survival and invasion levels and the presence of other virulence factors (i.e., *htrA*, *chuA*, and *lpfA*).

The analyses performed in this study, except those for colibactin and *htrA* (*p* = 0.044), indicated that there was no association between the virulence factors (*htrA*, *lpfA*, *chuA*, and *dsbA*) and cyclomodulin toxins (colibactin, *cnf1*, *cnf2*, and *cif*) (**Supplementary Table S5**).

### Dendrogram of the Rep-PCR Products From AIEC Isolates

**Figure 3:**
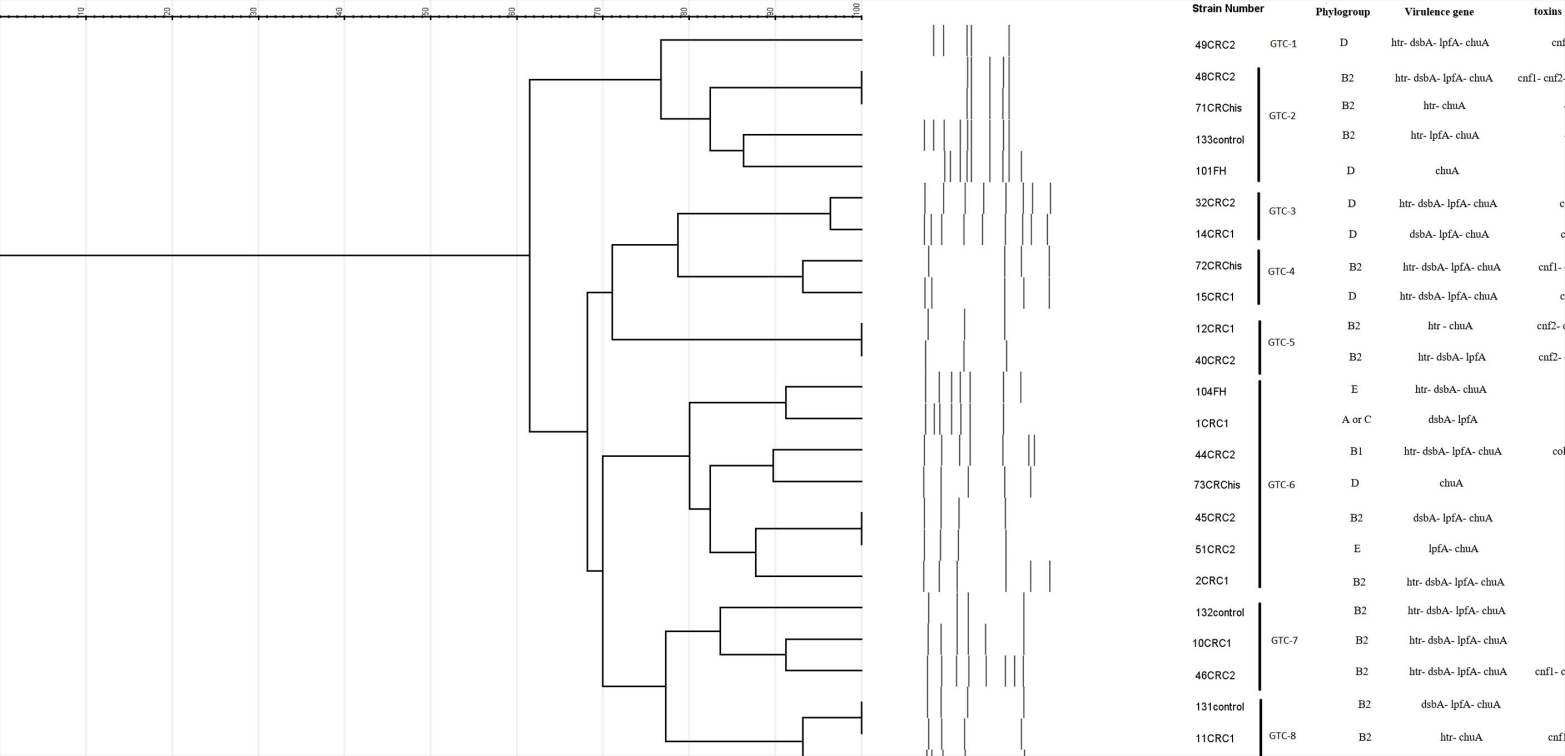
Dendrogram showing the genetic relatedness of the 24 adherent–invasive *Escherichia coli* (AIEC) strains determined by repetitive element sequence-based PCR (rep-PCR) analysis. Dice similarity coefficient and unweighted pair group method with average linkage clustering method were used (cutoff = 80%).

The dendrogram of the rep-PCR products from AIEC isolates is displayed in **Figure 3**. The counts of the bands ranged from 3 to 9, and the sizes of the fragments varied from 600 bp to 2 kb. Rep-PCR typing categorized the 24 AIEC isolates into eight GTC types according to the 80% cutoff. The most common type was GTC-6, which had seven isolates. In addition, four isolates were classified into genotype GTC-2, followed by genotype GTC-7 (three isolates), GTC-3, GTC-4, GTC-5, and GTC-8, each with two isolates, while one isolate showed a single type (**Figure 3**).

An overview of the phenotypic and genotypic features of the 24 AIEC isolates, including characterization of the studied groups, phylogroups, genotypes (rep-PCR), their virulence markers, and the adhesion, invasion, and macrophage survival values, is displayed in **Supplementary Table S6**.

## Discussion

Characterization of the intracellular AIEC examined in this study revealed that: i) this pathovar has higher adhesiveness, invasiveness, and survival capability in the cancer groups, particularly in the CRC2 group (comprising patients with tumors that have penetrated into the submucosal and deeper layers or T ≥1 stage) compared to the other groups; ii) B2 phylogroup intracellular AIEC isolates colonized in the tissue samples from the cancer groups were more frequent than those in the CRChis, FH, and control groups; iii) the genes that code for CM toxins were found to be overrepresented in the cancer groups, in particular *cnf1*, *cnf2*, and the colibactin gene; and iv) the left-sided colon carcinoma and adenocarcinoma T≥1 stage (CRC2) were colonized by B2 phylogroup AIEC-producing CMs more often than the samples from the other groups.

The results of this study showed that the mean age of the patients in the CRC1 group (55.03 years) was lower than that of patients in the CRC2 group (63.17 years). Advanced age is one of the significant risk factors for developing CRC (Hultcrantz, 2021). It occurs most frequently in people aged 50 years or older. A lot of CRCs can be prevented with regular screening. By screening for precancerous polyps, they can be removed before they become cancerous. CRC is usually asymptomatic in the early stages. Symptoms appear in the advanced stages of cancer (Ma et al., 2021). Hematochezia is a warning sign for CRC, but colon bleeding is sometimes very mild and cannot be detected. Therefore, FIT for occult blood helps in the diagnosis. As confirmed in this study, the majority of patients (40%) with a diagnosis of CRC had hematochezia or a positive FIT; therefore, these two factors can be warning signals for CRC.

Carcinogenic microbes can lead to genetic and epigenetic changes due to factors such as genotoxins, which are known to have carcinogenic effects (Lee et al., 2010; McCullough and Matson, 2016). Laboratory research indicated that specific microbes, including *E. coli*, enterotoxigenic *Bacteroides fragilis* (ETBF), *Streptococcus gallolyticus*, and *Fusobacterium nucleatum*, are associated with diseases such as CRC and are often driven by the intestinal microbiota (DeDecker et al., 2021).

Although a lot of studies have shown a high frequency of adherent *E. coli* strains in patients with CRC, little attention has been given to the presence of AIEC (Swidsinski et al., 1998; Martin et al., 2004; Buc et al., 2013; Bonnet et al., 2014). In this study, the frequency of intracellular *E. coli* isolates was higher within the studied groups, especially in the cancer groups (CRC1 and CRC2 groups), followed by the CRChis group; there was no significant difference in the frequencies of these isolates among the other groups. Furthermore, the properties of the investigated isolates revealed a significant difference in terms of adhesiveness and invasiveness between the groups, but not in terms of macrophage cell survival capability. The role of AIEC isolates in the pathogenesis of CRC has been demonstrated by numerous researchers (Buc et al., 2013). The results of this study indicated that the prevalence of AIEC in the cancer groups (CRC1 and CRC2 groups) was higher than that in the other groups.

Several researchers have found that family members have similar intestinal microbiomes, and given that the abundance of AIEC pathovars in patients with CRC is common, the frequency of this pathogen in individuals with a family history of CRC has been observed (Turnbaugh and Gordon, 2009; Lopez et al., 2021). It was hypothesized that individuals with a first-degree family history of CRC who are infected with AIEC might be more likely to be infected with this pathovar, whereas it was revealed that the frequency of AIEC in the FH group was almost the same as that in the control group. As shown in this study, AIEC was slightly isolated from healthy individuals in terms of its ability to exacerbate intestinal inflammation. Therefore, AIEC can be considered a possible pathogen in the pathophysiology of CRC. As seen in this study and in other studies, AIEC isolates have been observed, albeit to a lesser extent, in healthy individuals. Xu et al. found that bipolar pathobionts, such as AIEC, induce microbes to colonize their loci by inducing regulatory T cells (Tregs) and protecting the host against inflammation (Round et al., 2011). c-Maf and Stat-3 are required for the differentiation and function of microbiota-induced RORγt^+^ iTregs (induced Tregs). Moreover, microbial-specific iTreg *versus* nonspecific nTregs (natural Tregs) can also suppress inflammatory effector T cells (TEff) by better identifying similar epitopes (Xu et al., 2018). Meanwhile, the study by Iebba et al. showed that “bipolar” symbiotic AIEC may be involved in the high site substitution rate (SSR) of the FimH protein variants that can survive better in changing habitats (Iebba et al., 2012). According to Schippa et al., some genetic determinants, such as the altered expression patterns of various epithelial receptors, cause the gut microbiota to selectively prefer a particular habitat and increase the ability of AIEC strains to bind to the mucosa of patients with CRC and IBD (Schippa et al., 2009). Researchers have speculated that this pathovar may have a role in the progression of CRC. Pieces of evidence from *in vivo* studies have shown that AIEC can alter the intestinal microbiota in genetically sensitive hosts and spread a variety of pro-inflammatory microbes (Bretin et al., 2018). Thus, AIEC may be both the cause and effect of colorectal neoplasia.

As has been observed, although the frequency of AIEC isolates was higher in the CRChis group than that in the control group, the difference was not statistically significant. However, the AIEC isolates from the CRC groups (CRC1 and CRC2) showed much more invasiveness, adhesiveness, and survival capability compared to those from the other groups. There were significant increases in the adhesion, invasion, and survival values in AIEC isolates than in non-AIEC isolates.

The human large intestine is divided into different parts, and previous studies have shown that the microbial population of each part could be distinct (Hale et al., 2017). CRCs are characterized by their tumor area inside the colon (Stintzing et al., 2017). A right-sided colon carcinoma (RCC) includes the proximal two-thirds of the colon and cecum. The involvement of the distal third of the colon, splenic flexure, colon, sigmoid flexure, and rectum is termed left-sided colon carcinoma (LCC). In agreement with previous studies, our study showed that nearly 60% of the patients with CRC have LCC (Hemminki et al., 2010). This revealed that the primary location of the tumor in several patients in the cancer groups was the left-sided colon; in addition, most intracellular isolates (70.2%) and most AIEC isolates (62.5%) were isolated from LCC in all study groups. Accordingly, Buc et al. reported that LCC was more often colonized by B2 phylogroup *E. coli* producing CMs compared to RCC (Buc et al., 2013).

Phylogenic studies identified seven phylogenic groups for *E. coli* (A, B1, B2,C, D, E, and F) (Tenaillon et al., 2010; Clermont et al., 2013). The B2 and D groups mainly belong to pathogenic strains, whereas most normal flora strains are placed in groups A, C, and B1. Strains belonging to phylogroups B2 and D have virulence factors that are missing in the other groups (Tenaillon et al., 2010). In extraintestinal infections, most strains of B2 phylogroup *E. coli* are involved. A high frequency of B2 phylogroup *E. coli* is observed in patients with CRC, probably in terms of alterations in the host mucosal receptors in the mucosa-associated bacterial population. Studies have shown higher levels of B2 phylogroup *E. coli* colonization in IBD and in Crohn’s disease. In these patients, an increased intestinal CEACAM6 glycoprotein expression acts as a receptor for AIEC type 1 pili (Douaiher et al., 2017; Lee et al., 2019; Kamali Dolatabadi et al., 2021). CEACAM6 is a human tumor marker found in excess in colon tumors. Moreover, B2 phylogroup *E. coli* strains associated with Crohn’s disease intensify the expression of CEACAM6 receptors in gut epithelial cells (Jantscheff et al., 2003; Ferlizza et al., 2020; Yang et al., 2021). Most AIEC isolates belong to phylogroups B2 (54.2%) and D (29.2%). It was found that phylogroups A or C and B1 were the most abundant phylogroups (32% and 30%, respectively) in non-AIEC isolates. Previous studies have shown that most of the mucosal *E. coli* isolates belong to phylogroup B2 (Camprubí-Font et al., 2019; Lee et al., 2019).

According to the results of the genetic analysis of CM toxins, 54.16% of the AIEC isolates harbored carcinogenic genes. Colibactin and *cnf1* had the highest frequencies (29.2%), with *cnf2* and *cdt* having the second (25%) and third (4.2%) highest frequencies, respectively. Of the AIEC strains in the studied groups, 92.3% of those isolated from the cancer groups (CRC1 and CRC2) carried at least one of the CM toxin genes, while no toxin was detected in the FH and control groups. Moreover, 61.5% of the B2 phylogroup AIEC isolates carried CM toxins, whereas the A or C, E, and F phylogroup AIEC isolates did not carry any toxin. This finding is similar to those of other studies in which *pks*-positive *E. coli* was shown to be in excess in patients with CRC compared to non-cancer patients (Xie et al., 2016; Douaiher et al., 2017). It is likely that the generation of CM toxins can confer a particular advantage to the colonization of LCCs. This finding might be clarified by analysis of the microbiome composition and the physiology of the left-sided and right-sided colon. The epithelium of the digestive system within the left-sided colon primarily involves butyrate, while within the right-sided colon, acetic acid derivatives are more frequent. It has been suggested that bacteria are subjectively and quantitatively distinctive within the lumen of the right-sided and left-sided colon (Daniel et al., 2021). In this study, most of the CM-producing *E. coli* isolates included colibactin or a combination of the *cnf* gene and colibactin (46.13% of CM-producing *E. coli*), implying that colibactin plays an essential role in the dissemination of CM-producing *E. coli*. Nougayrède et al. suggested that the existence of *pks* encoding colitis could help colonize the gut tract (Nougayrède et al., 2006). Colibactin is a polyketide and non-ribosomal peptide (PK-NRP) complex that needs a number of various unique secondary metabolism-related precursors. Physiological differences between the left and the right colon can affect *E. coli* metabolism and, as a result, the synthesis of the PK-NRP complex into colibactin. Therefore, the distribution of CM-producing *E. coli* can be affected by the efficacy of colibactin. Proximal colon cancer can be caused by genomic instability linked with the microsatellite instability (MSI) phenotype (Wlaszczuk et al., 2022). Reciprocally, one of the main features of distal colon cancer is chromosomal instability (CIN) (Gervaz et al., 2002). *E. coli* carries colibactin, which can induce genetic instability and major chromosomal damage (Cuevas-Ramos et al., 2010). Furthermore, genes encoding *cnf1* and *cnf2*, which are mainly associated with colibactin, may cause abnormal chromosome cleavage. Therefore, these AIEC strains encoding CM may influence the development of CRCs.

It was observed by Bringer et al. that *dsbA* plays a major role in the pathogenesis of this pathovar, as the omission of *dsbA* in the AIEC LF82 strain led to pleiotropic results in the adhesion and subsequent invasion of gut epithelial cells through loss of the flagellum and type 1 pilus, as well as bacterial survival in macrophages. Moreover, they reported that the expression of *dsbA* and *htrA* is dependent on conditions of acidic and malnutrition stress caused by intramacrophagic bacteria. This rhythmic performance of the *htrA* and *dsbA* genes is beneficial for AIEC isolates because *htrA*, which is necessary for the macrophage proliferation of AIEC strains, should be modified after translation by DsbA (Bringer et al., 2005). Researchers are investigating other factors that can be modified after translation by *dsbA*, another regulator that plays a key role in regulating the intramacrophagic survival and replication of AIEC bacteria, which is needed for the production of most tumor necrosis factor alpha (Bringer et al., 2007). The *chuA* gene has an important role in obtaining iron and increasing the ability of AIEC to survive inside macrophages (Dogan et al., 2014). The *lpfA* gene was found in 68.4%–71% of AIEC strains and in 20%–28.1% of non-AIEC strains (Chassaing et al., 2011; Vazeille et al., 2016). The *lpfA*-free AIEC probably has virulence factors including *ibeA*, which contributes to their invasion into the epithelium. Four genes (*dsbA*, *htrA*, *chuA*, and *lpfA*) were identified with different distributions between the AIEC and non-AIEC strains. All of these virulence genes were discovered to be mainly related to AIEC strains rather than non-AIEC strains (*p* < 0.0001). It appears that AIEC strains carrying these virulence genes most likely belong to phylogenetic groups B2 and D and are capable of colonizing and surviving in epithelial cells and macrophages of patients with CRC.

Most of the isolates were genetically related based on the analysis of the rep-PCR banding profiles. In total, eight special GTC profiles were determined among the 24 AIEC isolates. In our observation, the most important type was GTC-6, into which 7 out of 24 (29.16%) AIEC isolates were grouped, and most of the isolates in this type were free of CM toxins and belong to the left facet of the colon. These findings might indicate that the isolation location and possibly the horizontal transfer of the CM toxin genes of the isolates are dependent on the clustering of the AIEC isolates.

## Conclusion

This research showed a high prevalence of intracellular CM generating B2 phylogroup AIEC in biopsies of patients with CRC and in individuals with a history of CRC compared to patients with a family history of CRC and the control group. The mean levels of adhesion, invasion, and intramacrophage survival of the AIEC isolates were significantly higher in the CRC groups than in others. Genetic relatedness was observed in the AIEC isolates with rep-PCR. The present findings are expected to provide inspirational insights into the possible role of intracellular CM producing B2 phylogroup AIEC in colon cancers. Large multicenter prospective clinical studies are needed to evaluate whether the detection of this pathovar can be a prognostic marker for CRC and whether therapeutic techniques that directly target AIEC, such as phage therapy, bacteriocins, and anti-adhesive compounds, can help in the prevention and treatment of CRC.

## Data Availability Statement

The original contributions presented in the study are included in the article/**Supplementary Material**. Further inquiries can be directed to the corresponding author.

## Ethics Statement

The studies involving human participants were reviewed and approved by Ethics Committee of Isfahan University of Medical Sciences. The patients/participants provided their written informed consent to participate in this study.

## Author Contributions

HF and MHE conceived and supervised the study. HF, RK, VK, and FM wrote the study protocol and data analysis plan. MHE, AF, and HR selected patients and obtained samples. RK processed the biopsies and performed microbiological and molecular analyses. HF, RK, and FM interpreted the data. RK drafted the manuscript. HF, VK, and FM revised the manuscript. All authors reviewed and approved the final manuscript, had full access to all the data in the study, and take final responsibility for the decision to submit the paper for publication.

## Funding

This study was supervised by HF and supported in part by a grant from Isfahan University of Medical Sciences (grant no. 398448, ethics code: IR.MUI.MED.REC.1398.319).

## Conflict of Interest

The authors declare that the research was conducted in the absence of any commercial or financial relationships that could be construed as a potential conflict of interest.

## Publisher’s Note

All claims expressed in this article are solely those of the authors and do not necessarily represent those of their affiliated organizations, or those of the publisher, the editors and the reviewers. Any product that may be evaluated in this article, or claim that may be made by its manufacturer, is not guaranteed or endorsed by the publisher.
