# Supplementary material for: Phenotypicand Genotypic Characterization of Clinical Isolates of Intracellular Adherent–Invasive Escherichia coli Among Different Stages, Family History, and Treated Colorectal Cancer Patients in Iran
Source: Front Cell Infect Microbiol. 2022 Jul 11;12:938477. doi: 10.3389/fcimb.2022.938477 (PMC9309365; doi:10.3389/fcimb.2022.938477)
Supplement: Supplementary file 1 [file DataSheet_1.docx]

Table1 Sup. Clinical features of individuals who took part in the trial.

FIT: Fecal immunochemical test; ND: No Data; CRC1: adenocarcinoma colorectal cancer in situ [Tis]; CRC2: colorectal cancer with tumor stage T1, T2 or T3; CRChis: CRC history, but normal colonoscopy results who have been referred for a 6-month follow-up; FH: normal results on colonoscopy, but with a family history of CRC based on National Comprehensive Cancer Network; ICisolates: intracellular isolate;

Sup Table2. Primers used for PCR analysis.

| Primer sequences(5'_3') | Target sequences | Amplicon size | Reference’s |
| --- | --- | --- | --- |
| virulence genes | | | |
| F-TGGTAATTACCGACGAAAACGGC  R-ACGCGTGGTTACAGTCTTGCG | uidA($\beta-$glucuronidase enzyme) | 162bp | (1) |
| F- TTCCAGCAGTTCTTCGGTGA  R-ATCAGTTCGCCGTTCAGGTT | htrA(stress protein) | 530bp | (2) |
| F- CTGGAAAACTGCGATATCTCC  R-GGCCTTCTTTCAGACGGTA | lpfA(long polar fimbriae) | 199bp | (2) |
| 5′-ATGGTACCGGACGAACCAAC-3′  5′-TGCCGCCAGTACCAAAGACA-3′ | chuA(Outer membrane hemin  receptor) | 288bp | (3) |
| cyclomodulin toxins | | | |
| F- GAAAGTAAATGGAATATAAATGTCCG  R- AAATCACCAAGAATCATCCAGTTA | cdtB(Cytolethal distending toxin, subunit B) | 467bp | (4) |
| F-GGCGACAAATGCAGTATTGCTTGG  R-GACGTTGGTTGCGGTAATTTTGGG | Cnf1(Cytotoxic necrotizing factor 1) | 552bp | (5) |
| F-GTGAGGCTCAACGAGATTATGCACTG  R-CACGCTTCTTCTTCAGTTGTTCCTC | Cnf2(Cytotoxic necrotizing factor) | 838bp | (5) |
| F- AACAGATGGCAACAGACTGG  R- AGTCAATGCTTTATGCGTCAT | Cif(cycle-inhibiting factor) | 383bp | (6) |
| F –GCGCATCCTCAAGAGTAAATA  R- GCGCTCTATGCTCATCAACC | ClbB(colibactinB) | 283bp | (1) |
| E. coli phylogenetic GROUP | | | |
| F-ATGGTACCGGACGAACCAAC  R-TGCCGCCAGTACCAAAGACA | chuA(Outer membrane hemin receptor) | 288bp | (7) |
| F-CAAACGTGAAGTGTCAGGAG  R-AATGCGTTCCTCAACCTGTG | yjaA(involved in the cellular response to hydrogen peroxide and acid stress) | 211bp |  |
| F-CACTATTCGTAAGGTCATCC  R-AGTTTATCGCTGCGGGTCGC | TspE4.C2(tsp encodes for a putative DNA fragment) | 152bp |  |
| F-AACGCTATTCGCCAGCTTGC  R-TCTCCCCATACCGTACGCTA | arpA(Ankyrin repeat protein A) | 400bp |  |

Sup Table3. Characteristics of the intracellular *E. coli* isolated strains

| Characteristics | | I-ADH^a^ | | | | I-INV^b^ (%) | | | | I-REPL^c^ (%) | | |  |
| --- | --- | --- | --- | --- | --- | --- | --- | --- | --- | --- | --- | --- | --- |
|  | | Mean±Std V | Median | IQR | pvalue | Mean±Std V | Median | IQR | pvalue | Mean±Std V | Median | IQR | pvalue |
| Refeence strain | E. coli  K-12 | 0.63±0.05 | - | - |  | 0.04± 0.03 | - | - |  | 25.09±4.34 | - | - |  |
|  | Shigella sonnei(ATCC 9290) | 3.64±1.13 | - | -- |  | 0.49± 0.304 | - | - |  | - | - | - |  |
|  | Salmonella enterica (ATCC 9270) | - | - | - |  | - | - | - |  |  |  |  |  |
| Disease group | CRC1(n=30) | 1.672±0.649 | 1.584 | 0.78 | 0.001 | 0.244±0.132 | 0.205 | 0.13 | <0.001 | 159.76± 146.044 | 74.82 | 197.98 | 0.124 |
|  | CRC2(n=30) | 1.607± 0.767 | 1.374 | 1.09 |  | 0.380± 0.217 | 0.3905 | 0.47 |  | 227.435± 242.174 | 86.1817 | 328.14 |  |
|  | CRChis(n=30) | 1.115± 0.211 | 1.030 | 0.31 |  | 0.163± 0.084 | 0.127 | 0.09 |  | 81.361± 58.418 | 78.888 | 73.60 |  |
|  | FH(n=30) | 1.212z± 0.256 | 1.220 | 0.39 |  | 0.113± 0.039 | 0.096 | 0.04 |  | 83.719± 40.884 | 69.675 | 38.94 |  |
|  | C(n=30) | 0.926± 0.278 | 1.004 | 0.07 |  | 0.097± 0.020 | 0.096 | 0.03 |  | 73.23± 38.934 | 57.140 | 68.62 |  |

Data was analyzed using No parametric Test ( Kruskal wallis) test. S, Significant (significant (<0.05*); NS, Not Significant for null (p >0.05, non-significant) association.

^a^ Grade of adhesion

^b^ Percentage of inoculum surviving after 1 h of gentamicin treatment (number of intracellular bacteria / initial inoculum x 100)

^c^ Number of intracellular bacteria at 24 h post-infection / number of bacteria at 1h post-infection x 100 (%)

CRC1: adenocarcinoma colorectal cancer in situ [Tis]; CRC2: colorectal cancer with tumor stage T1, T2 or T3; CRChis: CRC history, but normal colonoscopy results who have been referred for a 6-month follow-up; FH: normal results on colonoscopy, but with a family history of CRC based on National Comprehensive Cancer Network.

Sup Table4. Study of the relationship between virulence genes and invasion and survival levels

|  | | I-INV^a^ | Pvalue | I-REPL^b^ | Pvalue |
| --- | --- | --- | --- | --- | --- |
|  |  | Mean±Std V |  | Mean±Std V |  |
| lpfA | positive | 0.39±0.20 | 0.088(NS) | - | - |
|  | Negative | 0.24±0.12 |  |  |  |
| dsbA | positive | - | - | 359.79±207.31 | 0.015(S) |
|  | Negative |  |  | 147.77±45.98 |  |
| htrA | positive | - | - | 342.47±212.98 | 0.090(NS) |
|  | Negative |  |  | 189.83±116.85 |  |
| chuA | positive | - | - | 281.12±194.64 | 0.178(NS) |
|  | Negative |  |  | 483.10±229.78 |  |

Statistical significance of virulence genes and invasion and survival levels was calculated by One-way ANOVA test and association was determined. S, Significant (significant (<0.05*); NS, Not Significant for null (non-significant) association.

^a^ Percentage of inoculum surviving after 1 h of gentamicin treatment (number of intracellular bacteria / initial inoculum x 100)

^b^ Number of intracellular bacteria at 24 h post-infection / number of bacteria at 1h post-infection x 100 (%)

Sup Table5. Study of the relationship between virulence genes and cyclomodulins.

| virulence genes  cyclomodulins | | lpfA | | Pvalue | *htrA* | | Pvalue | *dsbA* | | Pvalue | *chuA* | | Pvalue |
| --- | --- | --- | --- | --- | --- | --- | --- | --- | --- | --- | --- | --- | --- |
|  |  | positive | Negative |  | positive | Negative |  | positive | Negative |  | positive | Negative |  |
| colibactin | Positive=7 | 5(71.4%) | 2(28.6%) | 0.967 | 7(100%) | 0 | **0.04** | 5(71.4%) | 2(28.6%) | 0.967 | 6(85.7%) | 1(14.3%) | 0.498 |
|  | Negative=17 | 12(70.6%) | 5(29.4%) |  | 10(58.8%) | 7(41.2%) |  | 12(70.6%) | 5(29.4%) |  | 16(94.1%) | 1(5.9%) |  |
| Cnf1 | Positive=7 | 6(85.7%) | 1(14.3%) | 0.303 | 6(85.7%) | 1(14.3%) | 0.303 | 6(85.7%) | 1(14.3%) | 0.303 | 7(100%) | 0 | 0.343 |
|  | Negative=17 | 11(64.7%) | 6(35.3%) |  | 11(64.7%) | 6(35.3%) |  | 11(64.7%) | 6(35.3%) |  | 15(88.2%) | 2(11.8%) |  |
| Cnf2 | Positive=6 | 5(83.3%) | 1(16.7%) | 0.437 | 6(100%) | 0 | 0.07 | 5(83.3%) | 1(16.7%) | 0.437 | 5(53.3%) | 1(16.7%) | 0.394 |
|  | Negative=18 | 12(66.7%) | 6(33.3%) |  | 11(61.1%) | 7(38.9%) |  | 12(66.7%) | 6(33.3%) |  | 17(94.4%) | 1(5.6%) |  |
| cdt | Positive=1 | 1(100%) | 0 | 0.512 | 1(100%) | 0 | 0.512 | 1(100%) | 0 | 0.512 | 1(100%) | 0 | 0.758 |
|  | Negative=23 | 16(69.6%) | 7(30.4%) |  | 16(69.6%) | 7(30.4%) |  | 16(69.6%) | 7(30.4%) |  | 21(91.3%) | 2(8.7%) |  |

Statistical significance of virulence genes and cyclomodulins was calculated by chi-square statistical analysis and association was determined. S, Significant (significant (<0.05*); NS, Not Significant for null (non-significant) association.

Sup table6. Phenotypic and molecular characterization of AIEC isolates collection.

| Reception number | Group disease | Phylogroup | cyclomodulin toxins | | | | | Virulence gene | | | | genotype (rep-PCR, | I-ADH^a^ (mean) | INV^b^ (mean) | REPL^c^ (mean) |
| --- | --- | --- | --- | --- | --- | --- | --- | --- | --- | --- | --- | --- | --- | --- | --- |
|  |  |  | *cnf1* | *cnf2* | *colibactin* | *cdt* | *cif* | *htrA* | *dsbA* | *lpfA* | *chuA* |  |  |  |  |
| 1(117) | CRC1 | A or C | - | - | - | - | - | - | + | + | - | GTC-6 | 1.72 | 0.63 | 320.62 |
| 2(72) | CRC1 | B2 | + | - | - | - | - | + | + | + | + | GTC-2 | 2.34 | 0.2 | 427.86 |
| 10(81) | CRC1 | B2 | - | + | - | - | - | + | + | + | + | GTC-7 | 2.49 | 0.22 | 519.55 |
| 11(107) | CRC1 | B2 | + | - | + | - | - | + | - | - | + | GTC-8 | 1.55 | 0.45 | 163.72 |
| 12(140) | CRC1 | B2 | - | + | + | - | - | + | - | - | + | GTC-5 | 2.07 | 0.22 | 244.37 |
| 14(40) | CRC1 | D | + | - | - | - | - | - | + | + | + | GTC-3 | 1.53 | 0.47 | 116.28 |
| 15(104) | CRC1 | D | - | + | - | - | - | + | + | + | + | GTC-2 | 1.62 | 0.19 | 338.00 |
| 40(15) | CRC2 | B2 | - | + | + | - | - | + | + | + | - | GTC-5 | 2.16 | 0.47 | 645.85 |
| 32(74) | CRC2 | D | + | - | - | - | - | + | + | + | + | GTC-3 | 1.92 | 0.41 | 322.21 |
| 44(122) | CRC2 | B1 | - | - | + | - | - | + | + | + | + | GTC-6 | 1.34 | 0.69 | 492.00 |
| 45(128) | CRC2 | B2 | - | - | - | - | - | - | + | + | + | GTC-6 | 2.07 | 0.37 | 394.48 |
| 46(158) | CRC2 | B2 | + | - | + | + | - | + | + | + | + | GTC-7 | 1.67 | 0.70 | 387.45 |
| 48(102) | CRC2 | B2 | + | + | + | - | - | + | + | + | + | GTC-4 | 3.81 | 0.68 | 478.67 |
| 49(145) | CRC2 | D | - | + | - | - | - | + | + | + | + | GTC-1 | 2.73 | 0.60 | 881.37 |
| 51(125) | CRC2 | E | - | - | - | - | - | - | - | + | + | GTC-6 | 1.41 | 0.39 | 114.16 |
| 71(52) | CRChi | B2 | - | - | - | - | - | + | - | - | + | GTC-2 | 1.41 | 0.16 | 119.53 |
| 72(159) | CRChi | B2 | + | - | + | - | - | + | + | + | + | GTC-4 | 1.55 | 0.18 | 187.05 |
| 73(43) | CRChi | D | - | - | - | - | - | - | - | - | + | GTC-6 | 1.38 | 0.38 | 116.27 |
| 74(95) | CRChi | D | - | - | - | - | - | + | + | - | + | GTC-8 | 1.02 | 0.24 | 187.00 |
| 101(53) | FH | D | - | - | - | - | - | - | - | - | + | GTC-2 | 1.72 | 0.13 | 138.70 |
| 104(103) | FH | E | - | - | - | - | - | + | + | - | + | GTC-6 | 1.38 | 0.13 | 176.37 |
| 131(18) | control | B2 | - | - | - | - | - | - | + | + | + | GTC-8 | 1.00 | 0.11 | 128.33 |
| 132(50) | control | B2 | - | - | - | - | - | + | + | + | + | GTC-7 | 1.05 | 0.37 | 113.69 |
| 133(55) | control | B2 | - | - | - | - | - | + | - | + | + | GTC-6 | 1.10 | 0.14 | 137.65 |

^a^ Grade of adhesion

^b^ Percentage of inoculum surviving after 1 h of gentamicin treatment (number of intracellular bacteria / initial inoculum x 100)

^c^ Number of intracellular bacteria at 24 h post-infection / number of bacteria at 1h post-infection x 100 (%)

CRC1: adenocarcinoma colorectal cancer in situ [Tis]; CRC2: colorectal cancer with tumor stage T1, T2 or T3; CRChis: CRC history, but normal colonoscopy results who have been referred for a 6-month follow-up; FH: normal results on colonoscopy, but with a family history of CRC based on National Comprehensive Cancer Network

**REFERENCES:**

1. Shimpoh T, Hirata Y, Ihara S, Suzuki N, Kinoshita H, Hayakawa Y, et al. Prevalence of pks-positive Escherichia coli in Japanese patients with or without colorectal cancer. Gut pathogens. 2017;9(1):1-8.

2. Prorok-Hamon M, Friswell MK, Alswied A, Roberts CL, Song F, Flanagan PK, et al. Colonic mucosa-associated diffusely adherent afaC+ Escherichia coli expressing lpfA and pks are increased in inflammatory bowel disease and colon cancer. Gut. 2014;63(5):761-70.

3. Nash JH, Villegas A, Kropinski AM, Aguilar-Valenzuela R, Konczy P, Mascarenhas M, et al. Genome sequence of adherent-invasive Escherichia coli and comparative genomic analysis with other E. coli pathotypes. BMC genomics. 2010;11(1):1-15.

4. Feng Y, Mannion A, Madden CM, Swennes AG, Townes C, Byrd C, et al. Cytotoxic Escherichia coli strains encoding colibactin and cytotoxic necrotizing factor (CNF) colonize laboratory macaques. Gut pathogens. 2017;9(1):1-15.

5. Sasaki M, Sitaraman SV, Babbin BA, Gerner-Smidt P, Ribot EM, Garrett N, et al. Invasive Escherichia coli are a feature of Crohn's disease. Laboratory investigation. 2007;87(10):1042-54.

6. Buc E, Dubois D, Sauvanet P, Raisch J, Delmas J, Darfeuille-Michaud A, et al. High prevalence of mucosa-associated E. coli producing cyclomodulin and genotoxin in colon cancer. PloS one. 2013;8(2):e56964.

7. Clermont O, Christenson JK, Denamur E, Gordon DM. The C lermont E scherichia coli phylo‐typing method revisited: improvement of specificity and detection of new phylo‐groups. Environmental microbiology reports. 2013;5(1):58-65.
